# Analysis of copy number variation using whole genome exon-focused array CGH in Korean patients with primary congenital glaucoma

**Published:** 2011-12-31

**Authors:** Ji Hyun Lee, Chang-Seok Ki, Hee-Jung Kim, Wool Suh, Seung-Tae Lee, Jong-Won Kim, Changwon Kee

**Affiliations:** 1 Department of Laboratory Medicine & Genetics, Samsung Medical Center, Sungkyunkwan University School of Medicine, Seoul, Korea; 2 Department of Ophthalmology, Samsung Medical Center, Sungkyunkwan University School of Medicine, Seoul, Korea

## Abstract

**Purpose:**

Primary congenital glaucoma (PCG) is an autosomal recessive form of glaucoma that manifests within the first year of life and if left untreated, leads to irreversible blindness. Cytochrome P450 1B1 (*CYP1B1*) is the major gene known to be associated with PCG. The role of the *CYP1B1* gene in disease pathogenesis and the relatively low detection rate of *CYP1B1* mutations in some populations, especially Asians, remain unexplained. We hypothesized that altered gene dosage of *CYP1B1* or anterior segmental dysgenesis causative genes may be involved in the pathogenesis of PCG.

**Methods:**

We performed whole genome exon-focused array comparative genome hybridization (aCGH) to identify copy number variation (CNV) in 20 Korean PCG patients and their parents.

**Results:**

We identified 12 patients with at least one rare gene-containing copy number variation each, corresponding to 25 CNVs (5 deletions and 20 duplications) at frequencies of 5-30% in PCG patients and 0% in controls. The 25 CNVs were not located at known chromosomal loci for PCG, namely GLC3A, which harbors *CYP1B1* (2p21), GLC3B (1p36.2-p36.1), or GLC3C (14q23), and did not include any target genes associated with PCG or anterior segmental dysgenesis.

**Conclusions:**

Further genetic studies with larger cohorts of patients are necessary to validate our results and to elucidate other genetic mechanisms underlying PCG, because the identified CNVs might be PCG-specific pathogenic variants and may explain the disease pathogenesis of PCG.

## Introduction

Primary congenital glaucoma (PCG), an autosomal recessive disorder of the eye that usually manifests within the first year of life, is caused by developmental defects in the trabecular meshwork and anterior chamber angle [1]. These developmental defects cause obstruction of aqueous outflow, leading to increased intraocular pressure and optic nerve damage. If left untreated, PCG leads to irreversible blindness [2].

Three loci for PCG (gene symbol GLC3), namely GLC3A (chromosome 2p2-p21), GLC3B (chromosome 1p36.2-p36.1), and GLC3C (chromosome 14q23), have been mapped by linkage analysis [3-5]. Cytochrome p450 1B1 (*CYP1B1*; OMIM 601771), which is associated with GLC3A, is known to be the major gene associated with PCG. The coding and promoter regions of *CYP1B1* have been screened extensively in PCG patients worldwide. However, the pathway by which *CYP1B1* affects development of the anterior chamber of the eye remains unknown. In addition, the prevalence of *CYP1B1* mutations varies in different populations, ranging from ~10% in Mexico to 100% in consanguineous Slovakian Gypsy patients. The incidence of *CYP1B1* gene involvement in Korean, Chinese, and Japanese PCG patients is relatively low (~20%), which means that it is not possible to explain the genetic pathogenesis of PCG in more than half of patients who do not have any *CYP1B1* mutations or only one mutant *CYP1B1* allele [6-10]. To try to explain these findings, we noted that anterior segmental dysgenesis (ASD) disorders such as Axenfeld-Rieger syndrome, Peters’ anomaly, and iris hypoplasia, are associated with raised intraocular pressure and an increased incidence of glaucoma. Furthermore, the eye is exquisitely sensitive to both reduced and increased doses of key developmental genes such as paired box 6 (*PAX6*) and forkhead box C1 (*FOXC1*), as demonstrated in ASD [11-15].

In this context, we expanded the disease spectrum of PCG to ASD and hypothesized that identification of underlying genomic imbalances could lead to elucidation of other genetic mechanisms underlying PCG. Therefore, our aim in this study was to explore alternative genetic mechanisms related to disease pathogenesis in PCG. We performed whole genome exon-focused array CGH (aCGH) to investigate the most susceptible copy number variations (CNVs) in 20 PCG patients and their parents. To the best of our knowledge, this is the first gene dosage analysis of PCG patients using aCGH.

## Methods

### Patients and clinical evaluation

Twenty Korean PCG patients and their unaffected parents were recruited during a multi-institutional collaborative study from September 2008 to February 2010 and included in this study. Clinical data including *CYP1B1* and myocillin (*MYOC*) sequencing results of subjects with PCG are shown in Table 1. Criteria for PCG diagnosis were evaluated when examination was possible and included IOP ≥21 mmHg in at least one eye; megalocornea; corneal edema/clouding/opacity; and glaucomatous optic nerve head damage. Corroborating features included symptoms of epiphora and photophobia. Patients with other ocular or systemic anomalies were excluded. Of the 20 PCG patients, only two patients had a known heterozygous mutation and a novel mutation in *CYP1B1* and *MYOC*, respectively.

**Table 1:** Clinical data including *CYP1B1* and *MYOC* sequencing results of subjects with primary congenital glaucoma.

**Individual number**	**Gender**	**Age of onset (months)**	**Affected eye(s)**	**IOP at diagnosis (OD;OS)**	**Family history**	***CYP1B1* mutation**	***MYOC* mutation**
1	M	10	U	22	-	-	-
2	M	3	B	22/16	-	-	-
3	M	76	B	30/24	Sibling	-	-
4	F	21	B	34/17	-	-	-
5	F	<1	B	30/32	-	-	-
6	M	29	U	34	-	-	L228S (heterozygote)
7	F	<1	U	23	-	-	-
8	F	<1	U	22.4	-	-	-
9	F	117	B	11/44	-	-	-
10	M	5	U	33/15	-	-	-
11	F	3	U	28	-	-	-
12	F	1	U	18.5	-	-	-
13	F	35	U	30	-	-	-
14	F	10	B	52/52	-	-	-
15	F	5	U	28	-	-	-
16	M	6	U	22.5		-	-
17	M	4	B	37/35	-	A330F (heterozygote)	-
18	M	2	B	26/32	-	-	-
19	M	10	U	18.5	-	-	-
20	F	1	B	21/30	-	-	-

Abbreviation: U, unilateral; B, bilateral.

### Array CGH analysis

To identify highly susceptible CNVs in PCG compared with control individuals, we performed aCGH on 20 PCG patients and their parents along with 99 healthy individuals. Genomic DNA was extracted from peripheral blood leukocytes using a Wizard Genomic DNA Purification kit according to the manufacturer’s instructions (Promega, Madison, WI). A total of 159 DNA samples were labeled and co-hybridized to determine DNA copy number changes in deletion/duplication using the NimbleGen Human CGH 3x720K Whole Genome Exon-Focused Array (Roche Diagnostics, Mannheim, Germany). Arrays were washed and then scanned using a NimbleGen MS 200 Microarray Scanner with 2 µm scanning resolution. Raw copy number data were normalized using Nexus software 5.0 (Nexus BioDiscovery, El Segundo, CA). All probe coordinates were mapped to the National Center for Biotechnology Information (NCBI) human genome assembly Build 36 (UCSC hg18). The normalized data were then processed with quality controls using Nexus Software and the manufacturer’s recommended default settings. To remove wave artifacts, we checked the number of aligned probes with signal quality and applied lowess correction to the log_2_ ratios. The same CNV types were merged with adjacent CNV calls using the criteria of ≤10 probes and ≤50 kb apart. The data were loaded into the Fast Adaptive States Segmentation Technique (FASST) segmentation algorithm with a significance threshold of 1.0×10^−5^. To minimize the number of false positive CNV calls without compromising the sensitivity of detection of true CNVs, we applied log_2_ ratios with thresholds of 0.3 in gain signals and −0.5 in loss signals. We visually inspected each sample, and excluded CNVs with <5 probes or CNVs ≤500 bp in length.

### Identification of copy number variations

**Figure 1:**
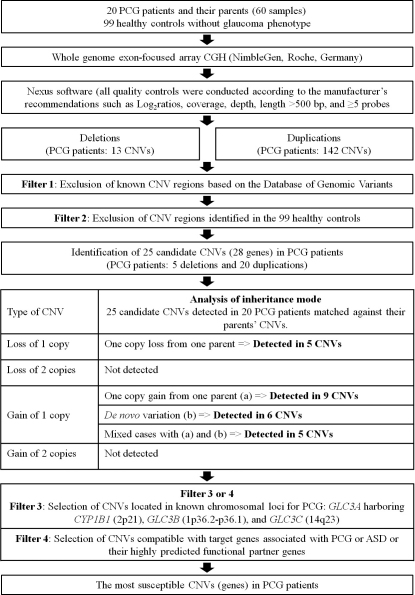
Summary of the copy number variation filtering strategy used in this study.

The CNV filtering strategy that we used is summarized in Figure 1. To describe the candidate CNVs, we used the Database of Genomic Variants (DGV; The Centre for Applied Genomics, Toronto, Canada) to determine whether the CNVs were novel or known. We excluded known CNVs in DGV and those identified in controls, and determined candidate genes using the identified CNVs.

We then matched the candidate genes detected in PCG patients against their parents’ CNVs to analyze the mode of inheritance. The identified CNVs were classified according to type (Figure 1) and we evaluated whether they were responsible for the disease phenotype of the patients or not.

To narrow down the potential candidate CNVs (genes) and match the identified CNVs to target regions and/or genes, we first focused on known chromosomal loci for PCG, namely GLC3A (2p2-p21), which harbors *CYP1B1*, GLC3B (1p36.2-p36.1), and GLC3C (14q23). Second, candidate genes with identified CNVs were matched against both PCG-related genes and ASD causative genes including *CYP1B1, MYOC, *latent transforming growth factor-beta binding protein 2 (*LTBP2*), *PAX6*, paired-like homeodomain transcription factor 2 (*PITX2*), *PITX3*, *FOXC1*, forkhead box E3 (*FOXE3*), eyes absent 1 (*EYA1*), LIM homeobox transcription factor 1 beta (*LMX1B*), and v-maf avian musculoaponeurotic fibrosarcoma oncogene homolog (*MAF*) [11,16-18].

**Figure 2:**
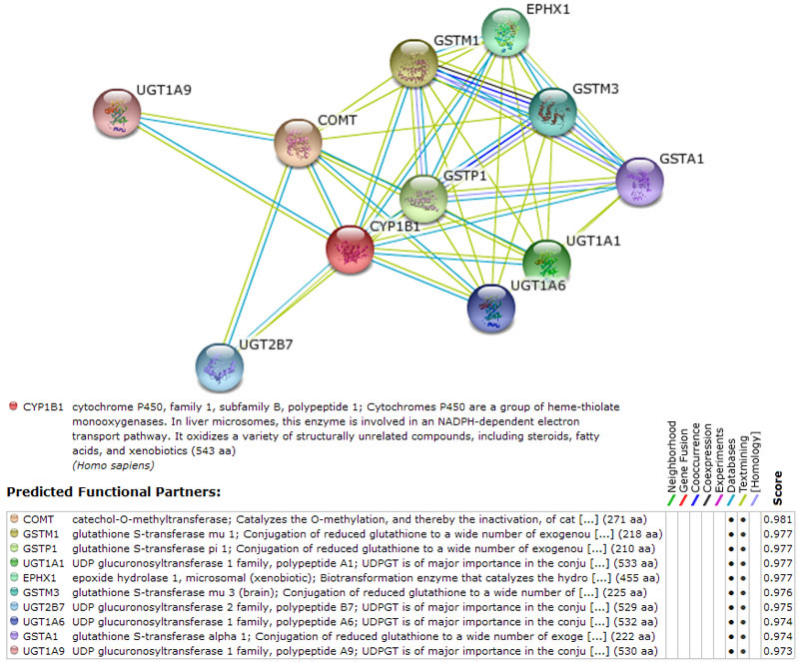
Evidence view of predicted functional partners of the *CYP1B* gene by STRING 8.3. Different line colors represent the types of evidence used to identify the associations.

We next identified highly predicted functional partners of the target genes listed above using the Search Tool for Retrieval of Interacting Genes/Proteins database (STRING, version 8.3), and we determined if any CNVs were associated with these genes (Table 2). The STRING database comprises known and predicted protein interactions, including direct (physical) and indirect (functional) associations; these interactions are derived based on genomic context, high-throughput experiments, conserved co-expression, and previous knowledge. An example of the STRING 8.3 results for *CYP1B1* is provided in Figure 2.

**Table 2:** Table of predicted functional partners of the *CYP1B1, MYOC, LTBP2, PAX6, PITX2, PITX3, FOXC1, FOXE3, EYA1, LMX1B*, and *MAF* genes by STRING 8.3.

***CYP1B1***	***MYOC***	***LTBP2***	***PAX6***	***PITX2***	***PITX3***
*COMT*	*OPTN*	*LTB*	*SIX3*	*CTNNB1*	*TH*
*GSTM1*	*CYP1B1*	*TGFBI*	*MITF*	*SUCLG1*	*SLC6A3*
*GSTP1*	*WDR36*	*ZNF185*	*SOX2*	*LEF1*	*GDNF*
*UGT1A1*	*SNCG*	*ELN*	*NEUROG2*	*FOXC1*	*NR4A2*
*EPHX1*	*FN1*	*LOXL1*	*SOX3*	*CCND2*	*MTA1*
*GSTM3*	*SERPINF1*	*MFAP2*	*IPO13*	*GIPC1*	*FOXE3*
*UGTB7*	*HRAS*	*COMMD9*	*PKNPX1*	*RGS1*	*SLC18A2*
*UGT1A6*	*TMTC1*	*ESYT3*	*TRIM11*	*RGS2*	*SOX2*
*GSTA1*	*TIMP1*	*ACYP1*	*SHH*	*RGS7*	*BDNF*
*UGT1A9*	*OLFM3*		*GCG*		*CHMP4B*
					
***FOXC1***	***FOXE3***	***EYA1***	***LMX1B***	***MAF***	
*PITX2*	*GPR161*	*SIX1*	*NPHS2*	*MYB*	
*DLL4*	*SOX2*	*SIX2*	*WNT7A*	*NPRL3*	
*HEY2*	*SMAD4*	*DACH1*	*NPS*	*NFE2*	
*ALX4*	*PITX2*	*PAX2*	*COL4A4*	*IL4*	
*MSX2*	*FOXF2*	*GDNF*	*PRODH2*	*ANPEP*	
*PAX6*	*FOXH1*	*SIX5*	*GPM6A*	*BACH2*	
*FGF2*	*SMAD2*	*PAX1*	*COK4A3*	*IRF4*	
*FLNA*	*HDAC9*	*TLX1*	*FOXA2*	*WHSC1*	
*CYP1B1*	*POU5F1*	*MYOG*	*LDB1*	*NFATC1*	
*CXCR4*	*RSRFR2*	*PAX3*	*LRP6*		

## Results

We performed aCGH to detect rare CNVs in PCG patients that were not present in 99 healthy controls. We identified 12 patients with at least one rare gene-containing deletion or duplication, corresponding to 25 CNVs (5 deletions and 20 duplications) at frequencies of 5%–30% in PCG patients and 0% in the controls (Table 3). According to the literature, most CNVs have a Mendelian inheritance pattern [19,20]. Therefore, we performed aCGH using parent-offspring trios, because the use of family information can improve the sensitivity and specificity of CNV detection. We matched 25 CNVs against the parents’ CNVs, which were classified into three main categories: loss of 1 copy from one parent (5 CNVs), gain of 1 copy from one parent (9 CNVs), and gain of 1 copy from de novo variation (6 CNVs). The remaining 5 CNVs were classified as ‘other’. No CNV was transmitted in an autosomal recessive inheritance manner. Considering that both parents of all patients were unaffected, loss or gain of one copy from only one parent is not likely to be associated with the disease phenotype. Only the gain of one copy due to de novo variation may represent an autosomal dominant mode of inheritance.

**Table 3:** Summary of 25 copy number variants in primary congenital glaucoma patients after the exclusion of known CNVs in the Database of Genomic Variants and the CNVs identified in 99 healthy controls.

**Case number**	**Frequency in case (%)**	**Chromosome location**	**Chromosome region**	**Size (bp)**	**CNV**	Mode of inheritance*	**Candidate gene(s)**
5	5.0	1p34.2	chr1:40,750,196–40,755,665	5470	loss of 1 copy	P	*DEM1*
16	5.0	8q22.1	chr8:95,459,849–95,465,598	5750	loss of 1 copy	M	*RAD54B*
7	5.0	12q24.33	chr12:131,802,886–131,811,641	8756	loss of 1 copy	P	*PGAM5*
6	5.0	15q21.2	chr15:50,254,727–50,267,146	12420	loss of 1 copy	P	*GNB5*
5	5.0	15q25.2	chr15:82,494,744–82,501,742	6999	loss of 1 copy	P	*ADAMTSL3*
5	5.0	1p33	chr1:47,463,023–47,466,352	3330	gain of 1 copy	D	*TAL1*
5, 11, 18	15.0	2p11.2	chr2:85,213,649–85,217,093	3445	gain of 1 copy	M, P/M, P	*TCF7L1*
15	5.0	2q24.1	chr2:158,089,932–158,132,486	42555	gain of 1 copy	P	*ACVR1C*
5	5.0	2q35	chr2:220,201,756–220,202,851	1096	gain of 1 copy	D	*SLC4A3*
5, 13	10.0	2q37.3	chr2:241,804,281–241,806,159	1878	gain of 1 copy	M, M	*ANO7*
18	5.0	6q14.1	chr6:79,840,645–79,848,461	7817	gain of 1 copy	P	*PHIP*
12, 13	10.0	7q32.1	chr7:127,456,885–127,463,642	6758	gain of 1 copy†	P, D	*LRRC4, SND1*
11, 12, 13, 14, 18	25.0	8q12.1	chr8:56,176,574–56,182,718	6145	gain of 1 copy†	P/M, M, D, D, P	*XKR4*
5	5.0	8q21.13	chr8:80,840,460–80,844,589	4130	gain of 1 copy	M	*HEY1*
5, 18	10.0	9q22.31	chr9:95,251,169–95,254,542	7827	gain of 1 copy†	M, D	*FAM120A, FAM120AOS*
3, 5, 10, 12, 13, 18	30.0	10q23.32	chr10:93,759,766–93,763,644	3879	gain of 1 copy†	P/M, M, D, M, M, D	*BTAF1*
3, 7, 12, 18	20.0	10q25.2	chr10:114,698,416–114,703,429	5014	gain of 1 copy	P/M, P, P/M, P	*TCF7L2*
5, 18	10.0	11q13.1	chr11:65,566,989–65,568,791	1803	gain of 1 copy†	M, D	*GAL3ST3*
12	5.0	11q13.1	chr11:65,868,765–65,875,077	6313	gain of 1 copy	M	*B3GNT1, BRMS1*
5	5.0	11q13.5	chr11:75,048,530–75,057,153	8624	gain of 1 copy	D	*MAP6*
7, 12, 18	15.0	11q23.3	chr11:120,535,111–120,539,731	4621	gain of 1 copy	M, M, M	*TECTA*
6, 12, 18	15.0	13q12.13	chr13:26,231,092–26,233,822	2731	gain of 1 copy	P, P/M, P/M	*GPR12*
14	5.0	16q21	chr16:56,778,172–56,788,677	10506	gain of 1 copy	D	*CSNK2A2*
12	5.0	16q23.1	chr16:73,587,930–73,593,272	5343	gain of 1 copy	D	*ZNRF1*
14	5.0	18q11.2	chr18:18,002,304–18,005,451	3148	gain of 1 copy	D	*GATA6*

Abbreviation: P, paternal; M, maternal; P/M, paternal or maternal; D, de novo. * Mode of inheritance is placed in order of case number. † Gain of one copy from one parent and gain of one copy de novo.

We found that the 25 CNVs, identified within 28 genes, were not located in known chromosomal loci for PCG, namely GLC3A (2p2-p21), GLC3B (1p36.2-p36.1), or GLC3C (14q23). These CNVs did not include any target genes associated with PCG or ASD, nor the predicted functional partners of the target genes, and none of the genes had a specific gene function that appeared to be relevant to the pathogenesis of PCG (Table 3).

These 25 identified CNVs might be PCG-specific pathogenic variants or may represent extremely rare benign variants that are not associated with the disease. Further genetic strategies to validate these CNVs are needed to identify specific gene functions relevant to the pathogenesis of PCG.

## Discussion

In this study, we performed aCGH in a series of 20 individuals diagnosed with PCG to discover novel copy number variations associated with this disease.

First, we chose target genes by expanding the disease spectrum of PCG to ASD, resulting in inclusion of the following target genes: *CYP1B1, MYOC, LTBP2, PAX6, PITX2, PITX3, FOXC1, FOXE3, EYA1, LMX1B*, and *MAF* [11,16-18]. Unlike PCG, ASD is classified into different subtypes according to the features of malformation affecting the anterior segment structure, e.g., aniridia, Axenfeld-Rieger syndrome, iridogoniodysgenesis, Peters’ anomaly, or posterior embryotoxon. However, some authors have claimed that PCG also involves abnormal development of Schlemm’s canal and trabecular meshwork drainage structures. Surprisingly, mutations in the ASD genes sometimes cause PCG, and PCG genes can also cause ASD [21,22]. In addition, dysregulation or mutation of a few ocular genes can cause a range of clinical conditions. For example, *PAX6* was first identified as the gene for aniridia, but is now known to underlie a range of other ocular conditions including Peters’ anomaly and a rare case of ASD [23-25]. *PITX2* and *FOXC1* mutations have been found in Peters’ anomaly as well as PCG [26-28]. Peters’ anomaly is also associated with mutations in two other genes, *CYP1B1* and the *FOXC1*-related gene, *FOXE3* [29]. Therefore, we hypothesized that PCG may be considered part of the ASD spectrum; common genetic pathways may underlie these two disorders.

We explored other genetic mechanisms underlying PCG by gene dosage analysis, because previous *CYP1B1* gene mutation studies have been unable to explain the allelic heterogeneity and pathogenesis of PCG in all patients. Previously, Kim et al. [10] performed direct sequencing analysis of all coding exons and flanking intronic regions of *CYP1B1* and *MYOC* in 85 Korean patients with PCG. These authors reported that about 70% of Korean PCG patients have neither *CYP1B1* nor *MYOC* mutations (*CYP1B1* mutation rate, 25.9%; *MYOC* mutation rate, 2.4%), results consistent with those reported for Japanese and Chinese patients. Indeed, 12 out of 22 patients had only one mutant allele in the *CYP1B1* gene [10]. In addition, the eye is known to be exquisitely sensitive to both reduced and increased gene dosage of key developmental genes. For example, gene dosage effects have been observed for *PAX6* and *FOXC1* in developmental ocular anomalies and ASD, respectively [12-15].

Overall, although we identified 25 CNVs (5 deletions and 20 duplications) in 12 PCG patients, we were unable to correlate these CNVs with the pathogenesis of PCG using a reference-based approach. The identified CNVs were not located in known chromosomal loci for PCG, namely GLC3A harboring *CYP1B1* (2p21), GLC3B (1p36.2-p36.1), or GLC3C (14q23), and did not include any target genes associated with PCG and ASD, nor highly predicted functional partners of target genes. However, our data suggest that altered gene dosage may explain the disease pathogenesis in PCG if these CNVs are PCG-specific pathogenic variants.

Our study has some limitations. We were unable to exclude the existence of pathogenic variants that were too small to be detected using our platform (<500 bp). Despite the high genomic resolution of aCGH used in our study, some genomic regions might not be covered well or may not have been assessed due to technical difficulties. Furthermore, we did not perform further studies to determine the gene functions or expression levels of the CNVs we identified.

In conclusion, this is the first study to comprehensively investigate gene dosage effects in PCG. We believe that the preliminary results and the CNV filtering strategy that we used can broaden our understanding of the genetic mechanisms underlying PCG.
